# Romanian consumer behaviour regarding traditional foods

**Published:** 2014

**Authors:** Purcărea Theodor Valentin, Orzan Gheorghe, Orzan Mihai, Stoica Ivona

**Affiliations:** * Romanian-American University, Bucharest, Romania, Bucharest Academy of Economic Studies, Bucharest, Romania

**Abstract**

Food communicates information, and food meaning depends on the social context in which food is found. The mode of preparation and consumption of food also expresses some cultural values. There is a high complexity of food behavior. For instance, consumers are affected by primary and secondary brand associations. Consumers are not all the same but they all need balanced food information, and to be reconnected with producers through adequate education and information about food production and preparation. Facing the challenge of a better dietary choice presupposes a proper change of food behavior. Within this framework, it is also challenging to see if people prefer traditional food and if they consider traditional food healthier than the processed food. And, when undertaking research of this nature, it is important to consider the relationship between the consumers' expectations, the consumer’s attitude to changes of traditional products and product acceptance, while also exploring the way attitudes towards innovation and contextual factors affect the appropriateness of traditional products.

**Keywords:** consumer behavior, traditional foods, production

